# VTX‐1463, a novel TLR‐8 agonist, attenuates nasal congestion after ragweed challenge in sensitized beagle dogs

**DOI:** 10.1002/iid3.91

**Published:** 2015-12-07

**Authors:** Christopher M. Royer, Karin Rudolph, Gregory N. Dietsch, Robert M. Hershberg, Edward G. Barrett

**Affiliations:** ^1^ Respiratory Immunology and Asthma ProgramLovelace Respiratory Research Institute2425 Ridgecrest Dr. SEAlbuquerqueNew Mexico87108USA; ^2^ VentiRx Pharmaceuticals, Inc.SeattleWashingtonUSA

**Keywords:** Allergic, dog model, nasal congestion, ragweed, rhinitis, rhinometry, toll‐like receptor

## Abstract

VTX‐1463 is a selective toll‐like receptor (TLR) 8 agonist that activates a subset of innate immune cells to produce a unique cytokine profile. Delivery of VTX‐1463 via nasal spray may modulate the nasal response in allergic rhinitis. The aim of this study was to determine the effects of VTX‐1463 on the nasal response in a dog model of allergic rhinitis. Ragweed (RW)‐sensitized dogs were pretreated with increasing doses of VTX‐1463 1 day prior to RW challenge or with two doses (4 or 8 days and 1 day prior to RW). Changes in nasal cavity volume (NV) were determined by acoustic rhinometry and nasal lavage fluid was assessed for histamine, lipid mediators, and cellular infiltrates at sequential times following RW challenge. VTX‐1463 pretreatment significantly preserved NV during the acute response to RW challenge for all doses tested. The area under the curve (AUC) for NV over the 1.5 h assessment period in RW challenged vehicle controls averaged 51.5% (SEM: ±2.12%) of the baseline NV over all studies. A single 100 µg dose of VTX‐1463 given 1 day prior to RW yielded an AUC for NV of 69.3% (±6.59%) of baseline, while a 1000 µg dose administered twice (8 days and 1 day prior to RW) resulted in an AUC for NV of 85.4% (±4.74%, *P* < 0.05) of baseline. For a single 1000 µg VTX‐1463 dose 1 day prior to RW, multiple mediators produced by mast cells, including histamine, PGE2, PGD2, and cysteinyl LTs, were significantly reduced relative to the vehicle control. The selective TLR8 agonist, VTX‐1463, preserved NV in a dose‐dependent manner in the acute phase of a nasal allergic response. The therapeutic effect appears to result from attenuated mast cell mediator release. Modulating the local cytokine response via TLR8 agonism appears to have a therapeutic effect on the acute allergic nasal response.

## Introduction

The chronic upper airway disease known as allergic rhinitis (AR) affects up to 42% of the population 1. Symptoms of AR arise from an immunoglobulin E (IgE)‐mediated reaction to allergen in the mucosa of the upper airways 2. In individuals with AR nasal, symptoms occur clinically as an early‐phase reaction, occurring within minutes of allergen exposure, and in up to 70% of patients followed by a late‐phase reaction, 4–8 h later 3. The early‐phase reaction results from basophil and mast cell degranulation in response to cross linking of allergen‐specific IgE bound to FcϵRI on the surface of the cells. Mast cells contain a number of proinflammatory and vasoactive mediators whose release to IgE mediated activation correlates to early‐phase symptoms in AR 3. These early‐phase mediators lead to a late‐phase response in which congestion becomes more prominent. Over time, a priming effect can develop, and decreased amounts of allergen are necessary for subsequent acute reactions to allergen 2. Managing the disease consists of controlling exposure to allergens and antagonizing the effects of known mediators, for example, by treatment with antihistamines, anti‐leukotrienes, and anti‐IgE antibodies, as well as control of inflammation and vasomotor responses 4.

Immunotherapy is currently the only therapeutic approach that can influence the natural history of atopic respiratory disease, and more recent approaches also hold the promise of affecting disease etiology 5. Since the discovery of the pattern recognition receptors of the innate immune system, it appears possible that modulating this system can be used to reprogram adaptive immune responses. Toll‐like receptors (TLRs) are a family of pattern recognition receptors, and selective agonists can be used to activate specific innate immune responses. Mediators produced in response to TLR stimulation can alter the development and maintenance of adaptive immune responses. Immune modulation using TLR agonists may facilitate a shift of responses away from common aeroallergens, thus providing a durable clinical benefit 5. Two TLRs implicated in allergy are TLR7 and TLR8, as studies have found variants in TLR7 and 8 genes contribute to genetic risk for atopic disease 6. However, TLR8 activation using selective agonists as an approach to modify allergic diseases including AR has received little attention. TLR8 is localized to endosomes where it recognizes single‐stranded RNA and is known to play an important role in immune activation to viral infection. However, the TLR8 cytokine response pattern could be used to modify immune responses to allergens. For example, by eliciting IL10 production and/or shifting the Th1/Th2 balance to allergens by modulating levels of Th1 polarizing cytokines such as IL12 and IFNγ 7, 8, 9.

Targeting TLR8 in allergic disease is facilitated by the availability of potent and selective small molecule (<500 MW) agonists. These agonists can be formulated for intranasal delivery and have chemical properties that allow them to effectively cross cell membranes and reach TLR8 that is localized in endosomal compartments. Immune response to this, class of compounds has considerable potential for pharmacotherapeutic modulation of TLR8‐derived responses. Here we present preclinical findings on the use of a novel small molecule TLR8 agonist, VTX‐1463, to modulate the nasal allergic response in our dog model 10 to demonstrate the therapeutic potential for the treatment of AR in humans.

## Materials and Methods

### Dogs

A total of 10 beagle dogs (two intact males and eight intact females) with preexisting nasal and airway allergic responses randomized into two treatment groups of 5, were utilized for the series of described studies. Early in life, these dogs were sensitized by subcutaneous (SC) injections of ragweed (RW), leading to the development of an antigen‐specific allergic immune response. Characteristics of the sensitization include eosinophilia in blood and lungs, a rapid and transient increase in pulmonary resistance following inhalation challenge with RW, as well as increased nasal congestion and inflammation following intranasal RW challenge 11. Dogs ranged in age from 7.5 to 9.5 years and body weights from 8.7 to 13.8 kg at the time of the study. All procedures involving animals were approved by the Institutional Animal Care and Use Committee of Lovelace Respiratory Research Institute.

## VTX‐1463

VTX‐1463 formulated in a vehicle of 25% Captisol® (Ligand Pharmaceuticals, Inc., La Jolla, California) in 10 mM citric acid solution was loaded into Accuspray™ Bi‐dose nasal delivery devices (Becton Dickinson, Franklin Lakes, NJ). The devices contained a 200 µl volume of either vehicle or VTX‐1463 dosing solution at a concentration needed to achieve doses of 100, 250, 500, and 1000 µg/dose (divided between nostrils, 100 µl/nostril). Nasal dosing of the vehicle and test compound was achieved by elevating the rostral muzzle and inserting a BD Accuspray device into the nares of alert dogs. The plunger of the spray device was depressed in a steady motion to dispense half the dose in the first actuation, and the process was repeated to deliver the second half of the dose into the contralateral nostril.

### Experimental design

Each dog served as its own control. Initially, all dogs were administered the vehicle control treatment, then challenged the following day with RW to establish a baseline response. Approximately 5 weeks later, dogs were randomized into two treatment groups of 5 (one male and four females each) based on their change in nasal cavity volume in response to RW challenge, thus ensuring a comparable response for the two groups. Baseline measurements of nasal cavity volume and nasal lavages before RW challenge were done 1 day prior to vehicle or VTX‐1463 pretreatment or prior to the first pretreatment if multiple treatments were evaluated. Post‐challenge measurements and lavages were done as listed below. RW extract (*Ambrosia artemisifolia*, Greer, Lenoir, NC; 6 mg/ml in 0.25 ml PBS) was instilled in both nostrils using an Accuspray device (Becton Dickinson). Single doses of vehicle and VTX‐1463 were given approximately 24 h prior to RW challenge (day 1). For studies assessing repeat VTX‐1463 treatments, the compound was given either 4 or 8 days prior (days 4 or 8, respectively) and again 24 h prior to RW challenge.

### Anesthesia

Dogs were anesthetized with isoflurane for all measurements and nasal lavages utilizing custom‐made face masks that cover the muzzle and provide an orally received breathing tube. This design for the induction of anesthesia limits isoflurane exposure of the nasal passages. After inducing general anesthesia, dogs were intubated and anesthesia was maintained with isoflurane throughout the experiment. Dogs were placed in dorsal recumbency for the nasal congestion measurements and in sternal recumbency with the head tilted to the side for the nasal lavage procedure. Heart rate, blood pressure, O_2_ saturation, and body temperature were measured and dogs were placed on a circulating water heating pad during anesthesia.

### Acoustic rhinometry

Nasal cavity volume (NV) was measured in anesthetized dogs using an Eccovision Acoustic Rhinometry System (Hood Laboratories, Inc., Pembroke, Massachusetts) as previously described 10. Briefly, a wave tube containing a spark sound generator was connected with the nasal cavity using a plastic nose piece. Based on nasal cast impressions and X‐ray measurements from the dog nasal cavity, a distance from the nostril opening into the nasal cavity of 10 cm was used for all experiments. Acoustic reflections were converted to area–distance function curves and used to determine nasal volume. Nasal cavity volume was measured before and at multiple time points following nasal RW challenge (both nostrils; 0, 15, 30, 45, 60, 75, and 90 min, 24 h, 48 h post‐RW challenge). Each measurement occurred immediately prior to any lavage of a given nostril collected at the same time point; however, only the nasal cavity volume from the non‐lavaged side was used for data analyses.

### Nasal lavage and analysis of mediator levels

While under anesthesia, a flexible plastic catheter was inserted 4–6 cm into the dog's nostril. The nostril was washed with a phosphate buffered saline solution. For mediator analysis, one 5 ml wash of one nostril was used at 0, 15, 30, 45, and 60 min following RW challenge. For determining cellular infiltration, three sequential 5 ml washes were collected at baseline, as well as 24 and 48 h post‐RW challenge. Nasal lavages of both nostrils to assess mediator levels (?) were performed at baseline (days −2, −6, −5, or −9, as well as 24 h prior to RW challenge), and at 24 and 48 h post‐RW challenge. Following RW challenge, only one nostril was lavaged for mediator analysis (15, 30, 45, and 60 min post challenge) to avoid compromising measurement of NV. Mediator levels in nasal lavages following RW challenge were compared to levels in the baseline lavage collected 24 h prior to a given treatment.

Total cell concentration in nasal lavage was determined using a Coulter Counter Analyzer (Model ZBI; Coulter Electronics, Hialeah, Florida). Cells were applied to slides by cytocentrifugation and stained with a modified Wright–Giemsa stain. A total of 400 cells were differentially counted based on morphology and staining characteristics and the percentage of each of four specific cell types (neutrophil, eosinophil, lymphocyte, and macrophage) were reported. Epithelial cells were excluded from these counts.

The supernatant of the lavage for mediator analysis was collected and stored separately at −80°C for batch processing. Samples intended for PGD2 assays were immediately processed as described in the instruction to convert PGD2 to a stable methoxime derivative (PGD2‐MOX) and samples after derivatization were frozen until used in the assay. Mediator analysis for histamine, leukotrienes, and prostaglandins was performed according to the kit manufacturer's instructions (Immunotech–Beckman, Indianapolis, IN, Coulter Company #IM2015, Neogen Corporation, Lexington, KY #406410, Cayman Chemical Company, Ann Arbor, MI: PGE2‐#514010, PGD2‐MOX‐#512011, respectively).

### Statistical analysis

Changes in nasal cavity volume, mediator levels, and cell counts over time comparing VTX‐1463 and vehicle pretreatment for each of the five experiments were assessed using two‐way analysis of variance (ANOVA) with Bonferroni post‐test (comparison at each time point). NV over the 1.5‐h period following RW challenge was fitted to a curve to assess the area under that curve (AUC). For each individual, a percent of the baseline AUC was calculated as the difference of AUC for a given treatment and the theoretical baseline maximum of 150 (100% × 1.5 h). Percent of baseline AUC values did not differ among vehicle treated groups and were averaged together. This value and that of each treatment was compared by ANOVA with the Bonferroni post‐test. *P* < 0.05 was considered significant.

## Results

### Evaluation of escalating single VTX‐1463 dose levels

**Figure 1:**
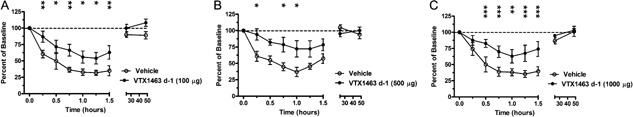
Time course for change in nasal cavity volume as a percent of baseline in response to RW challenge following vehicle or VTX‐1463 treatment as a single dose. Dogs received a single dose of 100 μg (A), 500 μg (B), or 1000 μg (C) of VTX‐1463 1 day prior to RW challenge. *P < 0.05; **P < 0.01, ***P < 0.001 relative to vehicle control for a given time point.

Single, increasing dose levels of VTX‐1463 resulted in a dose‐dependent improvement in the allergic response to RW. For the group of dogs pretreated with increasing VTX‐1463 doses, mean NV volume remained significantly closer to the baseline AUC determined over the1.5 h following RW challenge, than for the group of dogs pretreated with vehicle. The therapeutic effect of VTX‐1463 treatment was apparent during the early reaction to RW challenge based on NV. Within 15 min of the RW challenge, there was a marked decrease in mean NV in the vehicle‐treated dogs. With VTX‐1463 doses of 100 and 500 μg, the decrease in NV was significantly moderated for all time points over the 1.5‐h assessment period, (Fig. 1A and B). Pretreatment with a 1000 μg dose of VTX‐1463 produced a similar treatment effect, although the change in NV at 15 min did not achieved significance relative to the vehicle control (Fig. 1C). In both VTX‐1463 and vehicle‐treated groups, NV had returned to baseline levels by 24 h and remained near baseline 48 h post‐challenge.

In terms of maintaining a normal nasal cavity volume during the acute response (AUC of nasal volume over 1.5 h) to RW challenge, there was a clear dose‐related effect seen with VTX‐1463. When dogs were pretreated with 500 and 1000 μg doses of VTX‐1463, the decrease in NV was moderated and the difference was significantly better than for the vehicle controls. While the 100 μg dose of VTX‐1463 provided a small treatment effect, 69.31% of baseline versus 51.46% for vehicle controls, the improvement in NV did not achieve statistical significance (Table 1).

**Table 1:** Percent of baseline nasal cavity volume area under the curve over 1.5 h following RW challenge

Treatment	Vehicle	VTX‐1463 × 1	VTX‐1463 × 1	VTX‐1463 × 1	VTX‐1463 × 2	VTX‐1463 × 2
Dose	0 μg	100 μg	500 μg	1000 μg	250 μg	1000 μg
Dose day	−1	−1	−1	−1	−4, −1	−8, −1
Mean	51.46	69.31	81.40*	76.27*	80.70*	85.41*
SEM	2.12	6.59	7.69	7.16	5.50	4.74

Vehicle experiments did not differ and were averaged together for comparison with treated groups. Treatment groups did not differ from each other.

*P < 0.05 compared to vehicle treatment.

**Figure 2:**
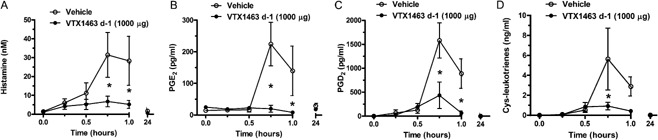
Histamine (A) and prostaglandins E2 (B), D2 (C), and cysteinyl leukotriene (D) analyses of serial nasal lavage samples in dogs treated with vehicle or a single dose of VTX‐1463 (1000 μg) 1 day prior to RW challenge. The same dogs were utilized by treating first with vehicle then 5 weeks later with VTX‐1463. *P < 0.05 relative to vehicle treatment for a given time point.

Mediators produced by activated mast cells were assessed in lavage fluid collected during the experiment assessing the efficacy of the 1000 μg dose of VTX‐1463 on NV. Lipid mediators and histamine levels were measured in serial nasal lavages from dogs treated with either vehicle or VTX‐1463, starting immediately after RW challenge. Large increases in mean histamine, PGE2, PGD2, and cysteinyl‐LT (cys‐LT) levels (Fig. 2A–D, respectively) were seen by 45 min in the vehicle group. Mean levels of all four mediators in lavage samples collected from the VTX‐1463 treated group were significantly lower at 45 min relative to vehicle‐treated controls. With the exception of CysLTs, the mediators in the VTX‐1463 group remained lower at 1 h post‐challenge lavage. All mediators returned to baseline values by 24 h post‐challenge for both vehicle‐ and VTX‐1463‐treated groups.

### Evaluation of multiple doses of VTX‐1463

**Figure 3:**
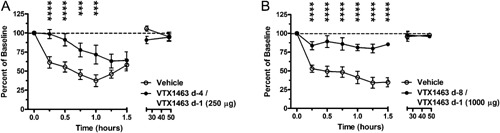
Time course for change in nasal cavity volume as a percent of baseline in response to RW challenge following vehicle or VTX‐1463 treatment as two doses. Dogs received two doses of 250 μg (A) or 1000 μg (B), 4 and then 1 day(s) prior (250 μg) or 8 and then 1 day(s) prior (1000 μg) to RW challenge. ***P < 0.001, ****P < 0.0001 relative to vehicle control for a given time point.

Based on the dose‐related improvement in NV achieved in the dog rhinitis model with a single VTX‐1463 dose, the efficacy of multiple pretreatments given prior to RW challenge was assessed. Two different pretreatment regimens were investigated: a 250 μg dose given twice on days −4 and −1, and a 1000 μg dose given twice on days −8 and −1. With RW challenge, the mean NV for VTX‐1463‐treated groups during the following 1.5‐h interval remained significantly closer to the baseline value than vehicle controls. Two VTX‐1463 doses consisting of either 250 μg or 1000 μg/dose significantly moderated the reduction in mean NV from 15 min though 1 h post‐RW challenge (Fig. 3A and B) relative to the vehicle control‐treated group. At the higher 1000 μg dose of VTX‐1463, the increase in NV remained significantly better than the vehicle group through 1.5 h post‐challenge (Fig. 3B). Nasal cavity volume returned to baseline levels by 24 h and remained so through 48 h post‐challenge for both VTX‐1463‐ and vehicle‐treated groups.

Comparing responses, two VTX‐1463 doses of 250 μg (4 days and 1 day prior to challenge) and two doses of 1000 μg (8 days and 1 day prior to challenge) were both significantly better than vehicle, based on changes in NV during the acute response to RW. Additionally, two 250 μg doses of VTX‐1463 given on days −4 and −1 provided a comparable therapeutic effect as single 500 and 1000 μg doses given −1 day prior to RW challenge (Table 1).

**Figure 4:**
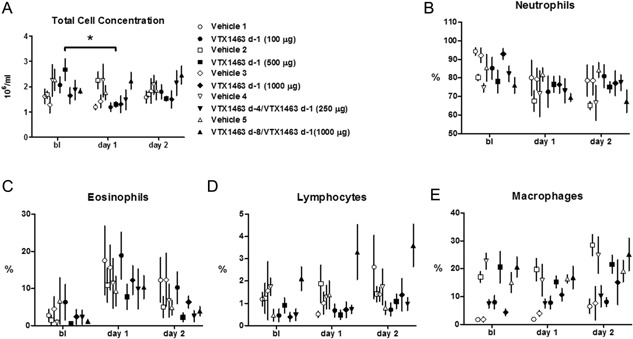
Total cell concentration in nasal lavage fluid (A) and percent of a total differential count for neutrophils (B), eosinophils (C), lymphocytes (D), and macrophages (E). Open symbols represent vehicle treatment while the respective closed symbol represents VTX 1463 treatment. Doses are indicated in the figure legend. The single dose treatment of VTX‐1463 (500 μg) led to a decrease from baseline in total cell concentration at day 1 (A; *P < 0.05). No other group differed from its respective baseline nor did any VTX‐treated group differ from the respective vehicle treatment and upon post hoc testing. See text for details.

In terms of cellular influx determined from nasal lavage fluid, VTX‐treated groups did not differ from the respective vehicle treatment and only a single group differed from its respective baseline upon post hoc testing; however, some overall differences were noted. The single dose treatment of VTX‐1463 (500 μg) led to a decrease in total cell concentration at day 1 (Fig. 4A). Neutrophils (Fig. 4B) decreased overall with time (*P* ****< 0.0001 by two‐way RM ANOVA). Eosinophils (Fig. 4C) increased overall from baseline (*P* ****< 0.0001 by two‐way RM ANOVA). Lymphocytes (Fig. 4D) differed among treatment groups (*P* ****= 0.0037 by two‐way RM ANOVA). Macrophages (Fig. 4E) differed among treatment groups (*P* ****= 0.0001 by two‐way RM ANOVA) and over time from baseline(*P* ****= 0.0009 by two‐way RM ANOVA).

## Discussion

A novel small molecule TLR‐8 agonist, VTX‐1463, attenuated the early‐phase reaction to nasal RW challenge in our dog model of AR. The effect of VTX‐1463 treatment was significant in the first minutes of the reaction that is largely histamine‐dependant 10, implying a mast cell stabilizing effect during allergen challenge. Further, the VTX‐1463 treatment induced attenuation of the proinflammatory mediators known to arise from mast cells (histamine, cys‐LTs, PGD2) that were found in serial nasal lavage samples following RW challenge (Fig. 2). Interestingly, this attenuation of an IgE‐mediated allergic reaction occurred by TLR agonism from as little as a single intranasal dose in the preceding 24 h. Our study complements two clinical trials in which the parent to VTX‐1463 improved nasal airflow and symptom scores following exposure to grass allergens 8.

To our knowledge, there is no evidence of TLR8 receptor expression by mast cells that would allow a direct modulation of the cells activity by selective agonists. However, other immune cells responding to TLR8 agonism may produce mediators that modulate mast cell activity. VTX‐1463 induces a unique Th1 cytokine and chemokine profile in PBMCs, including IL12, IFNγ, MCP‐1, MIP‐1β, as well as IL10 8, and this profile may contribute to the therapeutic effects observed in this study. IFNγ has been shown to inhibit the IL3 and IL4‐enhanced, IgE‐mediated serotonin and arachidonate release from rodent peritoneal mast cells 12, an effect arising primarily from IFNγ‐induced nitric oxide release that directly inhibits mast cells 13. The cytokine IL10 is a major modulator of the response to immunotherapy 14, and there are data to suggest it modulates the acute response to allergens in AR. It has been shown that the combination of IFNγ and IL10 production during venom immunotherapy decreases IgE‐mediated sulfidoleukotriene and histamine release of peripheral blood leukocytes 15. IL10 has also been shown to reduce mast cell density and degranulation in a rat model of acute myocarditis 16. Further, IL10 directly inhibits both cytokine and histamine release from human anti‐IgE activated, cord blood‐derived mast cells 17, an effect that fits the time course with which the apparent mast cell stabilization occurred in the present study. We were unable to measure cytokines in the current study, but other studies have demonstrated that selective TLR8 agonists induce IFNγ and IL10 in PBMCs 9. Overall, our findings, coupled with the published evidence described above 18, implicate IFNγ and IL10 as prominent players in this potential AR therapy.

Currently our data only provide information on the early phase efficacy of VTX‐1463 as the allergic response was well resolved by 24 h after allergen challenge in terms of nasal volume, histamine, and lipid mediators. In terms of cells identified in nasal lavage, eosinophils were highest at day 1 following allergen (Fig. 4C) but no changes due to VTX treatment were evident at the time points of this study. It appeared that a single dose of VTX1463 (500 μg) decreased the total cell concentration in nasal lavage at 24 h after allergen exposure (Fig. 4A); however, this was not a consistent finding. It will be important to further define the efficacy of VTX‐1463 as it temporally relates to allergen exposure. Interestingly, a single dose of VTX‐1463 given 24 h prior to allergen challenge attenuated the inflammatory mediator response and the decrease in nasal volume, an effect potentially due to the cytokines elicited as described above. TLR8 agonism has utility when administered prior to allergen exposure but given the relatively quick onset of action, TLR8 agonism may be useful as an adjunct to symptomatic treatment during exacerbations or for perennial AR patients. The clinical trials revealed some local adverse effects that may be addressed by optimizing the dosing strategy 8. Thus, further studies are needed to characterize the duration of action and efficacy following allergen‐induced exacerbation, as well as the potential long‐term effects of this therapeutic approach. The dog model appears to be well validated by the human clinical trials and should prove useful in these studies.

In summary, we have utilized an allergic dog model of AR to determine the efficacy of a novel small molecule TLR8 agonist. This approach allows direct application of the pharmacotherapy to the site of action in a clinically relevant large animal model. VTX‐1463 shows promise for the relief of the early phase nasal response to allergen; however, further studies are needed to determine the exact mechanisms of action, to optimize the dosing strategy, and to determine efficacy in the symptomatic patient and over the course of disease.

### Conflict of Interest

This study was performed at LRRI facilities and funded through a contract with VentiRx Pharmaceuticals. G. N. Dietsch and R. M. Hershberg were employees of VentiRx Pharmaceuticals at the time of this study.
